# Epithelioid angiosarcoma of the spine: A case report of a rare bone tumor

**DOI:** 10.3892/ol.2014.2055

**Published:** 2014-04-09

**Authors:** JUNZHE LANG, LEI CHEN, BI CHEN, KAI CHEN, AIHI LIU, JING LI, JING WANG

**Affiliations:** 1 Department of Orthopedics, The First Affiliated Hospital of Wenzhou Medical University, Wenzhou, Zhejiang 325000, P.R. China; 2 Department of Gynecology, The Second Affiliated Hospital of Wenzhou Medical University, Wenzhou, Zhejiang 325000, P.R. China

**Keywords:** epithelioid angiosarcoma, CD31, vertebral tumor, factor VIII-related antigen, spine

## Abstract

Epithelioid angiosarcoma (EA) is an extremely rare subtype of angiosarcoma, which is characterized by large cells with an epithelioid morphology. EA typically arises in deep soft tissues, including the adrenal gland, skin and thyroid, however, EA rarely arises in the spine. The current study presents a case of osteolytic lesions involving the fourth lumbar (L4) level of the spine. Preoperatively, the patient was misdiagnosed with metastatic carcinoma, however, a radiological examination detected the presence of osteolytic or destructive lesions in the vertebrae, which extended into the pedicles. Histopathological and immunohistochemical evaluations were performed on the tumor tissue obtained from a decompression specimen of the L4 vertebra. A bone lesion composed of sheet-like malignant cells exhibiting atypical epithelioid morphology with vascular formation was observed. The presence of anastomosing vascular channels lined by epithelioid endothelial cells also indicated that focal endothelial differentiation had occurred. In addition, immunohistochemistry assays revealed that the lesion was positive for the endothelial cell markers, CD31, CD34 and vimentin. The tumor was treated with decompression of the L4 vertebra, followed by posterior stabilization. The patient subsequently refused chemotherapy and radiotherapy but completed six months of follow-up. At the time of writing, the tumor remains under control and the patient is asymptomatic. This case highlights the difficulty of diagnosing EA, which requires careful pathological examination and immunophenotype labeling. At present, CD31 is the most sensitive marker for detecting EA.

## Introduction

Intraosseous epithelioid vascular tumors are generally classified as epithelioid hemangiomas (EHs), epithelioid hemangioendotheliomas (EHEs) or epithelioid angiosarcomas (EAs) (1–4). In addition, all of these tumor types may express the epithelial marker, cytokeratin, in addition to endothelial markers and vimentin (5). EA is a rare high-grade sarcoma of intraosseous vascular endothelial origin and is a rare variant of angiosarcoma. EA is characterized by rapidly proliferating, large polygonal epithelioid malignant cells with marked cellular pleomorphisms (6). As a result, the clinical/radiological presentation and cellular morphology of EA may be confused with multiple myeloma or metastatic carcinoma (5,7). Furthermore, focal areas of vasoformation and positivity for endothelial markers may lead to a misdiagnosis of other vascular bone tumors. EA has been found in the soft tissue, skin, adrenal gland, thyroid gland, vagina, uterus, breasts, lungs and gallbladder, but rarely in the bone (7–10). Thus, the presentation of EA in the spine is particularly rare. Consequently, the current study presents a case of a 76-year-old male with EA of the fourth lumbar (L4) vertebra. Patient provided written informed consent and the study was approved by the ethics committee of Wenzhou Medical University (Zhejiang, China).

## Case report

### Presentation and examination

**Figure 1:**
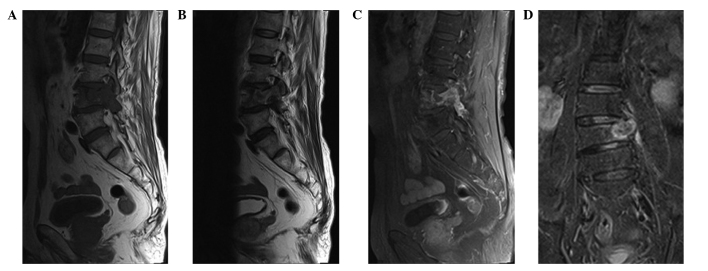
Magentic resonance imaging detected a cystic, destructive lesion in the L4 vertebra which exhibited a soft tissue component. (A) On T1-weighted sagittal image of the L4 vertebra, an intraosseous lesion with low signal intensity was observed and (B) on T2-weighted image, the lesion was slightly hyperintense. (C) Using short TI inversion-recovery imaging, the lesion exhibited high intensities. (D) On contrast-enhanced T1-weighted image, the lesion was homogeneously enhanced and was found to be compressing the adjacent spinal cord. L4, fourth lumbar.

**Figure 2:**
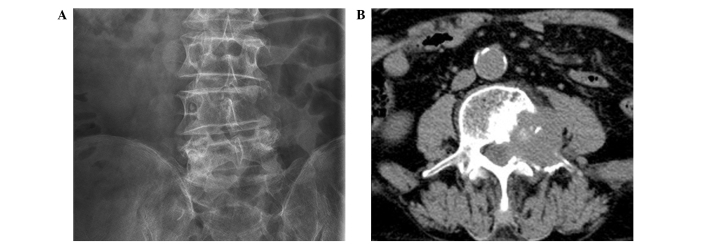
Expansile osteolytic lesion of the L4 vertebra, including erosion of the cortex. (A) A radiograph of the spine revealed an osteolytic lesion of the L4 vertebra involving erosion of the cortex. (B) Computed tomography scans of the lumbar spine revealed the presence of an expansive, lytic and destructive lesion with a cortical attenuation in the L4 vertebra which included a soft tissue component. L4, fourth lumbar.

In December 2013, a 76-year-old male presented to the Department of Orthopedics, The First Affiliated Hospital of Wenzhou Medical University (Wenzhou, China) with progressive pain over the mid-lumbar spine and a dull, aching and constant pain over the left side of the lumbar of five months. Subsequently, a root pain corresponding to the L4 dermatome was identified, which was aggravated by walking. A physical examination revealed the muscle strength and sensation to be weak in the left lower limb, and tenderness and percussion pain were present over the L4 vertebra. Magnetic resonance imaging (MRI) also detected a destructive soft tissue lesion occupying the L4 vertebral region, accompanied by moderate compression of the adjacent spinal cord which involved the pedicle and lamina. A knuckle deformity of the L4 vertebra was also observed, as well as cystic lesions present on T1-weighted images, which exhibited high intensities on T2-weighted images (Fig. 1). Computed tomography (CT) scans revealed the presence of an osteolytic lesion with ill-defined margins, which extended into the proximal soft tissue (Fig. 2). In addition, erosion of the cortex of the L4 was observed.

### Treatment

To decompress the spinal cord, a laminectomy of the L4 vertebra was performed. The lesion involved was 5 cm in length and 4 cm wide, and included the cystic and necrotic areas. The tumor was also poorly defined, had eroded the cortex and extended into adjacent soft tissue in an infiltrative pattern. A biopsy from the L4 vertebra revealed sections of softened bone, as well as areas of hemorrhage, necrosis and blood spaces; a phenotype distinctive of a red, hemorrhagic and friable tumor. In addition, a biopsy of the lesion was obtained.

### Histological examination

**Figure 3:**
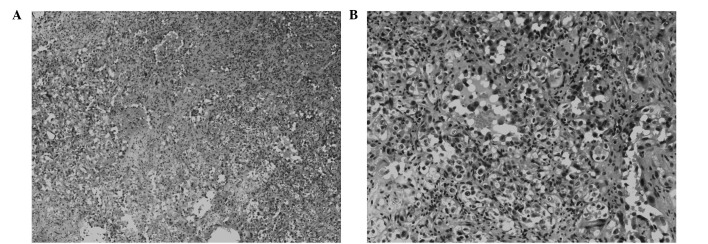
Proliferation of cells exhibiting epithelioid morphology, prominent nucleoli and abundant eosinophilic cytoplasm at magnifications of (A) ×40 and (B) ×100. Cells were accompanied by anastomosing vascular channels (stain, hematoxylin and eosin).

**Figure 4:**
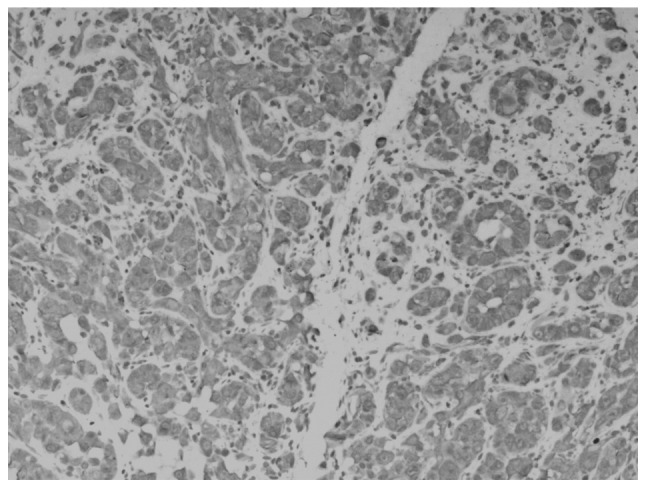
Expression of vimentin by tumor cells.

**Figure 5:**
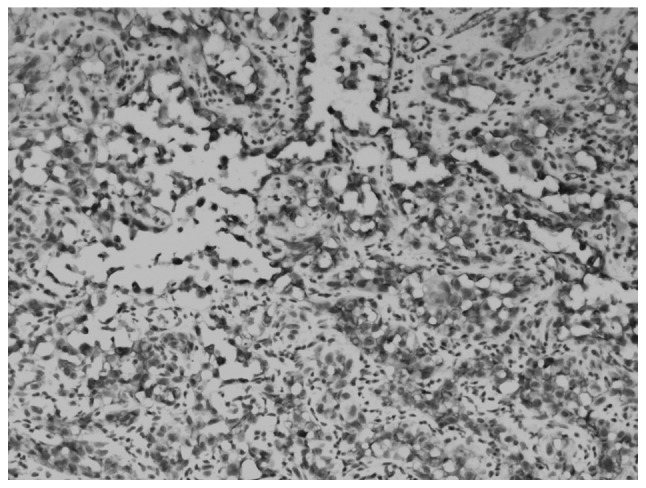
Expression of CD34 by tumor cells.

**Figure 6:**
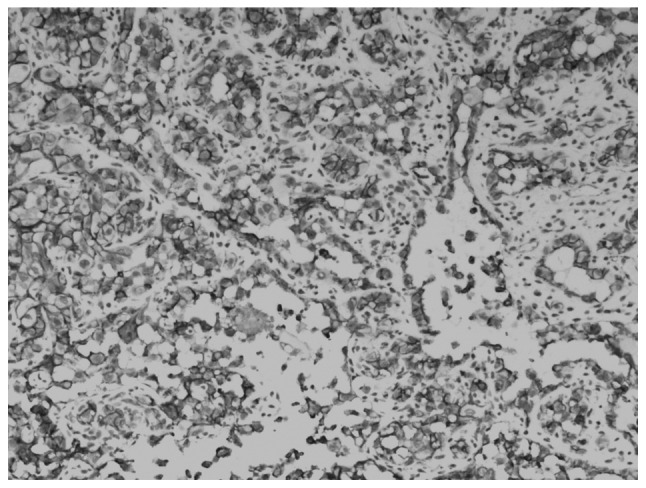
Expression of CD31 by tumor cells.

Hematoxylin-eosin staining was performed and sheets and nests of large, pleomorphic, round-to-polygonal epithelioid cells were observed. The cells exhibited abundant eosinophilic cytoplasm, vesicular nuclei and prominent nucleoli. Numerous brisk mitosis events exhibiting cellular pleomorphism were also observed and in certain areas, irregularly dilated vascular formations adjacent to the solid tissue were detected. Blood-filled channels were also found to be lined with epithelioid tumor cells, which provided a papillary appearance (Fig. 3). The stroma consisted predominantly of thin fibrovascular connective tissue, as well as varying degrees of hemorrhage, cystic changes and necrosis. Foci of prominent neutrophilic infiltrate were also observed (Fig. 3). Furthermore, in the immunohistochemistry assays, tumor cells were positive for vimentin (Fig. 4), CD34 (Fig. 5) and CD31 (Fig. 6) expression and negative for the epithelial membrane antigen (EMA). These results supported an endothelioid-related origin of the tumor.

### Postoperative course

The patient refused further therapy and was monitored for six months. At the time of writing, no evidence of tumor recurrence has been detected.

## Discussion

EA is a rare epithelioid vascular tumor of the bone, which accounts for <1% of all primary skeletal malignancies (6,11). The term EA refers to a variant of angiosarcoma which is characterized by tumor cells with an epithelioid morphology and these cells occasionally exhibit a pseudoglandular or alveolar arrangement (6,8). EA has previously been recognized in extraosseous sites, however, only a few cases of this rare variant have been documented in the bone (5,10). In addition, skeletal angiosarcoma tends to affect middle-aged and older individuals, has a marginal predominance for males and tends to affect the long tubular bones of the lower extremities, such as the femur and tibia (7,12). Literature regarding EA of the bone is limited to only a few case reports (6). Of these, Balicki *et al* (5) reported a case of multicentric EA in the two femurs of a 71-year-old patient and Marthya *et al* (13) reported a case of multicentric EA in the spine of a 65-year-old patient. Multicentricity has been observed in 20–50% of EA cases and consists of multiple lesions in a single bone, in the same extremity or throughout the skeleton. In CT and MRI scans, osseous destruction, central necrosis and marginal hyperperfusion of the soft tissue masses indicate the presence of an aggressive, high-grade bone tumor. However, the non-specific appearance of bone tumors may lead to a misdiagnosis of metastasis or multiple myeloma. Typically, EA positively stains for cytokeratins, including EMA and a variable proportion of EAs are positive for cytokeratin. In certain cases, a sheeted, epithelioid appearance is accompanied by positive cytokeratin staining. EA also closely mimics metastatic lesions and therefore, accurate diagnosis is difficult. Thus, an immunohistochemical examination of vasoformation may be informative and essential. In the present study, the tumor examined exhibited epithelioid morphology, with sheets and clusters of tumor cells characterized by prominent nucleoli and abundant eosinophilic cytoplasm. In addition, anastomosing vascular channels lined by layers of atypical endothelial cells were observed, which exhibited an anaplastic, immature appearance (8,10).

EA is associated with positive immunostaining for endothelial markers, including CD34 and CD31. Furthermore, CD31 is expressed by ~90% of angiosarcomas, but <1% of carcinomas and thus, CD31 is considered to provide a relatively high index of sensitivity and specificity (7,13). CD34 is also reported to be expressed by >90% of vascular tumors, however, this marker is much less specific and is expressed by several other soft tissue tumors (7). The immunoreactivity of factor VIII-related antigen can also be informative, although, it is variable for the diagnosis of bone EA. For example, it is often positive in epithelioid tumors, rather than conventional tumors. In addition, vimentin is a marker that is non-specifically expressed by all epithelioid vascular tumors (5). However, even in the absence of clear vascular differentiation, abundant intratumoral hemorrhage and the presence of intratumoral neutrophils are morphological changes that suggest a vascular origin (6,13).

With regard to the clinical behavior and prognosis of skeletal angiosarcoma, the majority of affected patients succumb to the disease within one year of diagnosis (5,6,13). Consistent with this poor prognosis, EA of bone is considered an aggressive high-grade tumor. The main differential diagnosis of EA of the bone includes the presence of other epithelioid vascular tumors, including EH, EHE or metastatic carcinoma (13). In addition, angiosarcoma of the bone usually presents with dull aching pain over the affected region (13) and the clinical course may progress to widespread and distant metastasis, which commonly affects lung and lymph node tissue (6). Radiological detection of EA is non-specific and typically includes an osteolytic lesion without periosteal reaction and soft tissue masses with cortical permeation (6). Furthermore, erosion of the cortex with soft tissue involvement may also be observed.

The most important differential diagnosis of EA is EHE, which is regarded as a vascular tumor of low-grade or borderline malignancy (14,15). While EHE shares a number of histopathological features with EA, EHE can be distinguished by the presence of pronounced, bland-appearing atypical cells, fewer cytoplasmic vacuoles, a lack of chondromyxoid and myxohyaline matrix, as well as a scarcity of endothelioid cells exhibiting cord-like growth and a less aggressive phenotype (12,13). Distinguishing EA from EHE and metastasis is important due to the significant differences in clinical treatment and prognosis of each (16). While the standard treatment for EA includes wide resection, chemotherapy and radiotherapy (6), an accurate diagnosis is required to avoid overtreatment.

EA and metastasis may involve multiple bone sites, affect older individuals and involve sheets of epithelioid tumor cells that tend to express keratin and EMA. As a result, a diagnostic bias may exist among pathologists and clinicians (17). Therefore, multicentric bone EA is easily misdiagnosed as metastatic carcinoma (18). However, metastatic lesions are almost always negative for the expression of CD34 and CD31, and lesions of EA are usually >5 cm in size (18). The latter is also associated with a significantly poorer prognosis. In the present study, the patient was initially misdiagnosed with metastatic carcinoma, however, vascular channels lined by atypical epithelioid cells and the immunohistochemistry results suggested a vasoformative tumor. In addition, the presence of an intratumoral neutrophilic infiltrate combined with the endothelioid nature of the cells confirmed a diagnosis of EA.

An additional differential diagnosis of EA is EH, which typically affects patients between the second and eighth decades of life, particularly those that are solitary. The long tubular bones are also most commonly affected by well-circumscribed lesions. Furthermore, well-formed vessels and a lobular growth pattern are characteristic of this tumor, in addition to an absence of severe nuclear atypia, which presents the benign nature of this tumor (13,19). By contrast, EHE is characterized by occasional grooves, fine chromatin, small nucleoli, mitotic counts of <5/10 high power fields and nuclear atypia. However, the latter presents to a lesser extent in EHE than compared with EA. An inflammatory component in the stroma, which is abundant in eosinophils and plasma cells also tends to accompany EHE (12,17).

Distinguishing EA from EHE, metastasis and EH is important due to significant differences in clinical behavior, treatment and prognosis for these conditions (13). In the English literature, no reports of EA presentation in the L4 region of the spine have been found. However, of the reported cases of EA, treatment usually includes wide resection, chemotherapy and radiotherapy. In the present study, treatment only included decompression of the L4 spine, as the patient refused further therapy. However, at the time of writing, six months following treatment, the tumor remains under control and the patient is asymptomatic. Furthermore, no evidence of tumor recurrence has been identified.

In conclusion, bone EA is rare and thus, the careful determination from EHE, EH and metastatic carcinoma is required due to differences in the management and clinical treatment for these conditions. The present study provides additional characterization of bone EA and emphasizes the utility of histopathological and immunohistochemical evaluations for its correct diagnosis, treatment and prognosis for individuals with this deceptive disease.

## Abbreviations

EA: epithelioid angiosarcoma
EHE: epithelioid heamangioendothelioma
EH: epithelioid hemangioma
MRI: magnetic resonance imaging
CT: computed tomography
